# Clinical-epidemiological characteristics and temporal trend of new cases of grade 2 disability leprosy in the state of Maranhão, Brazil, 2011- 2020

**DOI:** 10.1590/S2237-96222023000200026

**Published:** 2023-09-18

**Authors:** Rodolfo José de Oliveira Moreira, Janaína Miranda Bezerra, Floriacy Stabnow Santos, Lívia Maia Pascoal, Leonardo Hunaldo dos Santos, Marcelino Santos

**Affiliations:** 1 Universidade Federal do Maranhão, Programa de Pós-Graduação em Saúde e Tecnologia, Imperatriz, MA, Brazil

**Keywords:** Leprosy, Epidemiology, Incidence, Degree of Physical Disability, Time Series Study, Lepra, Epidemiología, Incidencia, Grado de Discapacidad Física, Estudio de Series Temporales, Hanseníase, Epidemiologia, Incidência, Grau de Incapacidade Física, Estudo de Séries Temporais

## Abstract

**Objective:**

to describe the clinical and epidemiological characteristics of new cases of grade 2 disability leprosy and to analyze its trend in the state of Maranhão, from 2011 to 2020.

**Methods:**

this was a descriptive cross-sectional and ecological time-series study, using data from the Notifiable Health Conditions Information System. A descriptive analysis of the event was carried out according to the sociodemographic and clinical-laboratory characteristics of the cases. The temporal trend of event incidence was analyzed using Prais-Winsten regression.

**Results:**

of the 2,147 cases, 71.5% were male, 48.9% had up to 8 years of schooling, 66.5% were of mixed race/color, 95.5% had the multibacillary form, 58.8% were borderline, 32.3% had negative bacilloscopy at diagnosis. There was a stationary trend in the state and a falling trend in the São Luís Health Region (annual percentage change = -64.4%; 95% confidence interval: -73.7;-51.9).

**Conclusion:**

incidence trend was stable in the state of Maranhão and falling in São Luís.

## Study contributions

**Main results:**

Out of 2,147 grade 2 disability leprosy cases, the majority were male, of mixed race/skin color, multibacillary and borderline. The São Luís regional health unit showed a falling trend.

**Implications for services:**

The results can guide strategies for the leprosy control program in the state, aiming at new approaches towards early diagnosis, treatment and prevention of disabilities.

**Perspectives:**

Further studies are needed, such as spatial distribution of cases and detection rates of leprosy in children under 15 years of age, in order to gain a better understanding of the epidemiological profile of leprosy in Maranhão.

## Resumen

**Objetivo:**

describir las características clínicas y epidemiológicas de los nuevos casos de lepra con discapacidad física grado 2 y analizar su tendencia en el estado de Maranhão, de 2011 a 2020.

**Métodos:**

estudio transversal descriptivo y ecológico de serie temporal con datos del Sistema de Información de Agravamiento de Notificaciones. Se realizó un análisis descriptivo de características sociodemográficas y clínico-laboratoriales. La tendencia temporal de la incidencia de eventos se analizó mediante la regresión de Prais-Winsten.

**Resultados:**

de los 2.147 casos, 71,5% era masculino, 48,9% tenía hasta 8 años de escolaridad, 66,5% era de raza/piel parda, 95,5% de forma multibacilar, 58,8% dimorfa, 32,3% con baciloscopia negativa al diagnóstico. Hubo tendencia estacionaria en el estado y tendencia decreciente en la Región de Salud São Luís (variación anual = -64,4%; intervalo de confianza 95%: -73,7;-51,9).

**Conclusión:**

la tendencia de la incidencia se mantuvo estable en el estado de Maranhão y decreciente en São Luís.

## Resumo

**Objetivo:**

descrever as características clínico-epidemiológicas dos casos novos de hanseníase com grau 2 de incapacidade física e analisar sua tendência no estado do Maranhão, Brasil, 2011-2020.

**Métodos:**

estudo transversal descritivo e ecológico de série temporal, com dados do Sistema de Informação de Agravos de Notificação; realizou-se análise descritiva do evento segundo características sociodemográficas e clínico-laboratoriais dos casos; a tendência temporal da incidência do evento foi analisada pela regressão de Prais-Winsten.

**Resultados:**

dos 2.147 casos notificados, 71,5% foram do sexo masculino, 48,9% possuíam até 8 anos de estudo, 66,5% eram de raça/cor da pele parda, 95,5% da forma multibacilar, 58,8% da forma dimorfa e 32,3% com baciloscopia negativa no diagnóstico; observou-se estacionaridade na tendência no estado, e tendência decrescente na regional de saúde de São Luís (variação anual = -64,4%; intervalo de confiança de 95% -73,7;-51,9).

**Conclusão:**

a tendência da incidência foi estável no estado do Maranhão e decrescente em São Luís.

## INTRODUCTION

Leprosy is a chronic communicable disease; and it is neglected despite representing a public health problem in Brazil and worldwide. It is an infection of the skin and peripheral nerves, with granulomatous characteristics, and which has, as an etiological agent, the *Mycobacterium Leprae* bacillus.^1^ ^),(^ ^2^ Also known as Hansen’s bacillus, it has an affinity for peripheral nerves and their infection can result in nerve damage with permanent physical disabilities, especially in the eyes, hands and feet, if there is no specific form of treatment.^3^

Leprosy cases can be classified according to the number of skin patches or lesions and grade of disability (GD).^4^ ^),(^ ^5^ A person with grade 2 disability (G2D) has significant impairment, visible in the eyes, hands and feet.^3^

At the global level, in 2019, the rate of G2D leprosy diagnosis was 1.4 case/1 million inhabitants, reaching a total of 10,816 worldwide.^6^ That was when the World Health Organization (WHO) established the goal of reducing rate of new cases with G2D to less than 1 case/1 million inhab., to be achieved by 2020.^6^

In Brazil, also in 2019, 2,351 new cases of G2D leprosy were reported, corresponding to an incidence rate of 11.2 cases/1 million inhabitants.^8^ In that year, the G2D incidence rate in the state of Maranhão was 31.5 new cases/1 million inhab., accounting for 223 cases of G2D leprosy.^7^ In view of data demonstrating late diagnosis of leprosy and active transmission of the disease,^4^ ^),(^ ^7^ the need to understand G2D leprosy diagnosis is justified in Maranhão, throughout the 2010s, especially.

The objective of this research note was to describe the clinical and epidemiological characteristics of new cases of leprosy with G20 and to analyze its trend in the state of Maranhão, Brazil, from 2011 to 2020.

## METHODS

This was both a descriptive cross-sectional study, aimed at the clinical-epidemiological characterization of new G2D leprosy cases, and also an ecological study, regarding the analysis of temporal trend in detection of new G2D cases in Maranhão, between 2011 and 2020. Maranhão comprises 217 municipalities and has a population of 7,153,262 inhab.^8^

The ecological units analyzed were the state’s regional health units (RHU). The state of Maranhão is divided into 19 RHU: Açailândia, Bacabal, Balsas, Barra do Corda, Caxias, Chapadinha, Codó, Imperatriz, Itapecuru Mirim, Pedreiras, Pinheiro, Presidente Dutra, Rosário, Santa Inês, São João dos Patos, São Luís, Timon, Viana, and Zé Doca.^9^

The data used for the analysis were extracted from the Notifiable Health Conditions Information System (SINAN) on September 28, 2021, from database downloads in .cvs format files. The compulsory leprosy notification forms provide clinical and sociodemographic data, which were used as variables in this study.^10^ Leprosy cases with G2D at the time of diagnosis in the state of Maranhão in the period 2011-2020 were selected.

The variables selected for the descriptive analysis were:

a) sex (male; female);

b) age (at last birthday: 0-14; 15-29; 30-49; 50-59; 60 and over);

c) race/skin color (White; Black; Asian; mixed race; Indigenous; unknown);

d) schooling (illiterate; up to 8 years of schooling; over 8 years of schooling; not applicable; unknown);

e) clinical form (indeterminate; tuberculoid; borderline; virchowian; not classified; unknown);

f) operational class (multibacillary; paucibacillary);

g) bacilloscopy at diagnosis (positive; negative; not performed; unknown); and

h) number of lesions present (single lesion; 2-5; more than 5; not informed).

A descriptive analysis of case frequency distribution (absolute and percentage) according to sociodemographic and clinical-laboratory characteristics was performed. The new G2D leprosy case detection rate was obtained by dividing the number of new cases of G2D leprosy by the population residing in the area, in the same period, multiplied by 1 million.^4^ The trend in new G2D case detection was classified as rising, falling or stable, according to the Prais-Winsten regression coefficient value: a positive value indicates a rising trend; a negative value means a downward trend; and a null value or p-value without significance means a stable trend.^11^ In order to test the statistical difference in the trend, annual percentage change (APC) was estimated, along with a 95% confidence interval (95%CI) and a 5% significance level. APC represents the average rate of change in disease incidence over a year, for each of the identified trends.^11^ SPSS 24.0 software was used for statistical treatment of the data.

The study project was exempted from submission and appraisal by a Research Ethics Committee, since it was based exclusively on public domain data.

## RESULTS

We assessed 2,147 cases of individuals diagnosed with leprosy and G2D treated in the state of Maranhão, within the proposed time frame.

Regarding case description, the majority were male (71.5%) and of mixed race/skin color (66.5%), in the multibacillary operational class (95.5%) and with the borderline clinical form (58.8%). Also noteworthy were the schooling of those affected with up to 8 years of complete study (48.9%), presence of more than 5 lesions (48.4%), age above 60 years (33.2%) and negative bacilloscopy at diagnosis (32.3%), as shown in Table 1.

**Table 1:** Clinical and epidemiological characteristics of new leprosy cases with G2D at diagnosis/year, Maranhão, Brazil, 2011-2020

Variables	N	%
Sex		
Male	1,536	71.5
Female	611	28.5
Age (at last birthday)		
≤ 14	104	4.9
15-29	376	17.5
30-49	604	28.1
50-59	351	16.3
> 60	712	33.2
Race/skin color		
White	321	14.9
Black	334	15.6
Asian	18	0.9
Mixed Race	1,429	66.5
Indigenous	6	0.3
Unknown	39	1.8
Schooling (in completed years)		
Illiterate	557	25.9
≤ 8	1,051	48.9
> 8	313	14.6
Not applicable	5	0.3
Unknown	221	10.3
Operational class		
Multibacillary	2,050	95.5
Paucibacillary	97	4.5
Clinical form		
Indeterminate	44	2.1
Tuberculoid	102	4.8
Borderline	1,250	58.8
Virchowian	543	25.5
Not classified	162	7.6
Unknown	26	1.2
Bacilloscopy at diagnosis		
Positive	626	29.2
Negative	694	32.3
Not performed	680	31.7
Unknown	147	6.8
Number of lesions		
Single lesion	193	9.0
2-5	498	23.2
> 5	1,039	48.4
Not informed	417	19.4
Total	2,147	100.0

The new G2D case detection rate was 35.2 cases/1 million inhab. in 2011, and 15.7/1 million inhab. in 2020 (Table 2). The new G2D case detection trend in the state proved to be stationary in the period studied (APC = -27.4%; 95%CI -53.3;13.0; p-value = 0.150) and in almost all the RHU : the exception was the São Luís RHU, where a falling G2D trend was found (APC = -64.4%; 95%CI -73.7;-51.9; p-value < 0.001).

**Table 2:** Rate of new leprosy cases with G2D^a^ at the time of diagnosis (per 1 million inhab.), distributed by regional health units, Maranhão, Brazil, 2011-2020

RHU^b^	2011	2012	2013	2014	2015	2016	2017	2018	2019	2020	Annual Percentage Change % (95%CI^c^)	p-value	Status
Açailândia	51.1	53.9	70.7	24.4	37.9	47.8	30.4	33.9	33.6	23.3	-6.9 (-22.4;11.7)	0.410	Stable
Bacabal	42.1	99.2	83.8	61.0	74.6	74.5	45.2	40.8	37.0	14.8	-5.2 (-17.6;9.2)	0.420	Stable
Balsas	42.6	14.0	27.7	4.5	9.0	26.9	17.8	31.1	53.0	13.1	-3.2 (-17.2;13.2)	0.660	Stable
Barra do Corda	9.2	9.1	40.5	13.4	21.9	17.4	21.6	21.5	17.1	8.5	-0.5 (-22.1;27.1)	0.970	Stable
Caxias	31.1	37.6	40.6	37.0	26.7	19.9	19.8	49.2	22.8	19.5	-4.9 (-26.0;22.1)	0.660	Stable
Chapadinha	11.2	13.9	19.4	24.6	18.9	10.7	10.6	21.3	21.1	2.6	-7.5 (-38.4;38.8)	0.680	Stable
Codó	39.8	29.7	42.6	68.6	39.0	29.2	35.5	31.9	50.9	41.3	0.2 (-19.4;24.7)	0.980	Stable
Imperatriz	30.9	36.4	26.6	26.5	43.3	15.0	18.6	31.4	38.6	11.0	-5.4 (-24.0;17.7)	0.580	Stable
Itapecuru Mirim	37.1	36.5	21.8	29.6	26.6	28.9	31.2	23.5	38.9	20.6	-9.4 (-34.4;25.1)	0.510	Stable
Pedreiras	37.2	55.8	41.7	50.8	51.2	60.5	23.2	32.0	31.9	22.8	-5.8 (-24.7;17.8)	0.560	Stable
Pinheiro	18.5	10.5	2.6	7.7	12.8	15.3	5.0	25.2	5.0	5.0	-6.2 (-31.0;27.5)	0.640	Stable
Presidente Dutra	14.3	53.4	28.3	38.8	35.1	10.5	31.4	37.9	34.3	10.2	-1.1 (-16.7;17.4)	0.890	Stable
Rosário	7.2	3.5	3.5	3.4	17.1	3.3	16.7	3.3	9.9	3.2	-1.8 (-32.2;42.1)	0.910	Stable
Santa Inês	88.9	80.3	98.0	81.6	53.0	58.0	91.0	41.2	30.8	12.7	-8.6 (-18.1;2.0)	0.120	Stable
S. João dos Patos	21.3	21.1	21.0	25.0	8.3	16.5	8.2	24.4	28.4	4.0	-7.3 (-29.7;22.1)	0.560	Stable
São Luís	47.4	51.9	43.9	42.0	45.1	33.5	40.8	34.6	39.8	25.2	-64.4 (-73.7;-51.9)	< 0.001	Falling
Timon	21.2	12.6	0.0	16.5	28.6	28.4	4.0	28.1	24.0	11.9	-1.6 (-22.1;24.4)	0.880	Stable
Viana	15.6	0.0	11.4	11.3	26.2	33.5	22.2	18.4	21.9	3.6	-3.6 (-28.4;29.4)	0.790	Stable
Zé Doca	43.0	42.4	17.4	10.3	27.2	10.1	30.1	30.1	33.2	13.2	-7.3 (-24.7;14.1)	0.430	Stable
Maranhão	35.2	38.1	35.4	32.8	34.3	28.4	29.4	30.4	31.6	15.7	-27.4 (-53.3;13.0)	0.150	Stable

a) G2D: Grade 2 disability; b) RHU: Regional health unit; c) 95%CI: 95% confidence interval.

## DISCUSSION

Detection of new G2D leprosy cases showed a stable trend for state of Maranhão as a whole, while the São Luís RHU showed a falling trend. In the state of Maranhão, this form of infection has been hyperendemic. Besides causing serious physical limitations to the individual, G2D can revive stigmas and social prejudices associated with the disease for thousands of years.^4^ ^),(^ ^6^ ^),(^ ^7^

A higher proportion of leprosy cases with G2D was identified in males. A hypothesis has been raised that the higher frequency of late diagnosis of the disease in males is due to the lower number of men seeking care in health services.^12^ Another hypothesis for this finding would be testosterone itself, the predominant male sex hormone, which stimulates the T helper 2 immune response, which is the main response in the multibacillary class, in which G2D is more predominant.^13^

Leprosy cases with G2D were predominant in individuals aged over 60 years. Leprosy is a chronic disease and degree of disability is related to the time course of morbidity. In the elderly individual, therefore, the chances of physical disabilities and their severity are greater.^14^ ^),(^ ^15^

This analysis showed a higher frequency of G2D cases in individuals with low schooling. There is evidence that lower levels of schooling contribute to a decrease in self-care and a reduction in the demand for care in health services, favoring the transmission of leprosy and the development of physical disabilities.^16^ ^)-(^ ^18^

There was a higher proportion of cases classified as multibacillary and with the borderline clinical form. The multibacillary classification, which the borderline form falls into, is identified as a risk factor for physical disabilities. This aspect of multibacillary classification and diagnosis of physical disability points to late diagnosis.^19^ ^),(^ ^20^

Regarding bacilloscopy, although there were fewer positive results when compared to negative ones, the risk of cases of leprosy with positive bacilloscopy at diagnosis presenting physical disabilities related to leprosy is two times greater.^21^

There was a higher proportion of G2D leprosy cases with more than 5 lesions. The presence of more than 5 lesions in a person with leprosy is a characteristic of the multibacillary operational classification, which is a risk factor for the development of physical disabilities due to the disease.^4^ ^),(^ ^19^ ^),(^ ^20^

There was stability in the detection trend of new cases of leprosy with grade 2 physical disability, in almost all RHU in the state of Maranhão; the only exception was the São Luís RHU, where this trend was found to be falling. The stationary trend in the state of Maranhão as a whole and the falling trend in São Luís can be attributed to the actions of the Family Health Strategy and its teams in the fight against leprosy, which has been in place since 1994.^22^ From the year 2000 onwards, the effectiveness of the Family Health Strategy in Maranhão led to an increase in the number of diagnosed leprosy cases and, consequently, an increase in all related indicators, followed by a progressive drop in their rates.^22^

As limitations of this study, we highlight (i) possible underreporting of leprosy in the state of Maranhão and (ii) possible errors in filling out notification forms, given that the analysis includes only secondary data, which could compromise, mainly, the description of the clinical-epidemiological profile of the cases. Errors in filling out data are due to factors such as health worker lack of knowledge and overburdening, in addition to information system shortcomings.^14^

We conclude that this study described the clinical and epidemiological profile of new cases of leprosy with G2D in the state of Maranhão, whereby there was a predominance of males, mixed race/skin color, age over 60 years, up to 8 years of schooling, multibacillary classification and borderline clinical form, as well as negative bacilloscopy at diagnosis. A stable trend was identified for G2D leprosy in the state, in general, while a falling trend was only found for the São Luís RHU. We recommended reinforcing active tracing aimed at achieving early diagnosis as the best approach to leprosy, in the sense of early diagnosis, treatment and prevention of disabilities.
